# Earliest Porotic Hyperostosis on a 1.5-Million-Year-Old Hominin, Olduvai Gorge, Tanzania

**DOI:** 10.1371/journal.pone.0046414

**Published:** 2012-10-03

**Authors:** Manuel Domínguez-Rodrigo, Travis Rayne Pickering, Fernando Diez-Martín, Audax Mabulla, Charles Musiba, Gonzalo Trancho, Enrique Baquedano, Henry T. Bunn, Doris Barboni, Manuel Santonja, David Uribelarrea, Gail M. Ashley, María del Sol Martínez-Ávila, Rebeca Barba, Agness Gidna, José Yravedra, Carmen Arriaza

**Affiliations:** 1 IDEA (Instituto de Evolución en África), Museo de los Orígenes, Madrid, Spain; 2 Department of Prehistory, Complutense University, Madrid, Spain; 3 Department of Anthropology, University of Wisconsin-Madison, Madison, Wisconsin, United States of America; 4 Institute for Human Evolution, University of the Witwatersrand, Johannesburg, South Africa; 5 Plio-Pleistocene Palaeontology Section, Department of Vertebrates, Ditsong National Museum of Natural History (Transvaal Museum), Pretoria, South Africa; 6 Department of Prehistory and Archaeology, University of Valladolid, Valladolid, Spain; 7 Archaeology Unit, University of Dar es Salaam, Dar es Salaam, Tanzania; 8 Department of Anthropology, University of Colorado Denver, Denver, Colorado, United States of America; 9 Department of Anthropology, Complutense University, Madrid, Spain; 10 Museo Arqueológico Regional, Alcalá de Henares, Madrid, Spain; 11 CEREGE (Centre Européen de Recherche et d'Enseignement des Géosciences de l'Environnement) Aix-Marseille Université (AMU/CNRS/IRD/Collège de France), BP80, Aix-en-Provence, France; 12 CENIEH (Centro Nacional de Investigación sobre la Evolución Humana), Burgos, Spain; 13 Department of Geodynamics, Complutense University, Madrid, Spain; 14 Department of Earth and Planetary Sciences, Rutgers University, Piscataway, New Jersey, United States of America; Illinois State University, United States of America

## Abstract

Meat-eating was an important factor affecting early hominin brain expansion, social organization and geographic movement. Stone tool butchery marks on ungulate fossils in several African archaeological assemblages demonstrate a significant level of carnivory by Pleistocene hominins, but the discovery at Olduvai Gorge of a child's pathological cranial fragments indicates that some hominins probably experienced scarcity of animal foods during various stages of their life histories. The child's parietal fragments, excavated from 1.5-million-year-old sediments, show porotic hyperostosis, a pathology associated with anemia. Nutritional deficiencies, including anemia, are most common at weaning, when children lose passive immunity received through their mothers' milk. Our results suggest, alternatively, that (1) the developmentally disruptive potential of weaning reached far beyond sedentary Holocene food-producing societies and into the early Pleistocene, or that (2) a hominin mother's meat-deficient diet negatively altered the nutritional content of her breast milk to the extent that her nursing child ultimately died from malnourishment. Either way, this discovery highlights that by at least 1.5 million years ago early human physiology was already adapted to a diet that included the regular consumption of meat.

## Introduction

**Figure 1:**
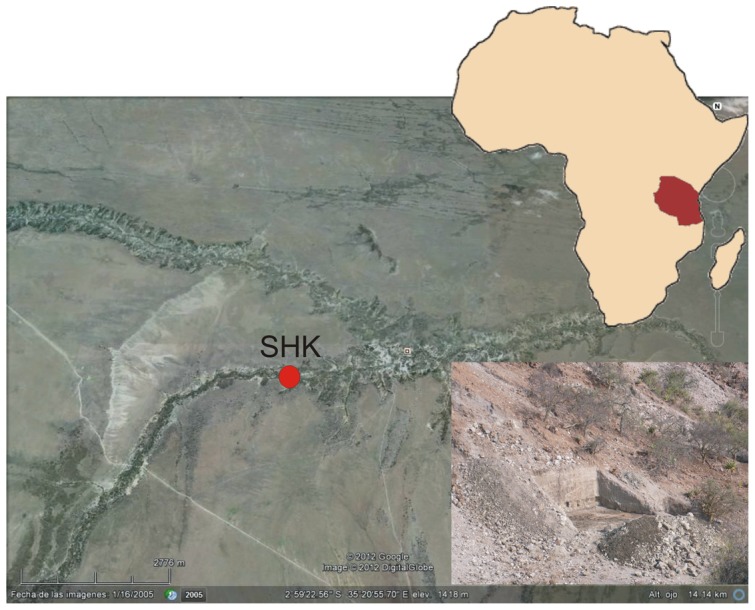
Aerial view of Olduvai Gorge (Tanzania) with the approximate site of SHK indicated. The inset shows the current excavations at the site.

We report the discovery of porotic hyperostosis on Olduvai Hominid (OH) 81, two refitting right parietal fragments of a ∼2-year-old child (Hominidae gen. et sp. indet.) from the 1.5-million-year-old (Ma) SHK (Sam Howard Korongo) site, Olduvai Gorge, Tanzania (Fig. 1). Porotic hyperostosis is a bone pathology associated with anemia [1], [2]. Cranial bones affected by porotic hyperostosis show thinning or obliteration of the vault's outer table, as well as lesions that seem to emanate from the hypertrophy of the diploë [3]. Porotic hyperostosis has been observed rarely on the bones of modern human foragers [4] on only a few Middle (KNM-ES 11693) and Upper Pleistocene (Villabruna 1; Magdalenian) fossils of early *Homo sapiens* [5], [6], and never before on the fossils of hominins from the early Pleistocene record.

**Figure 2:**
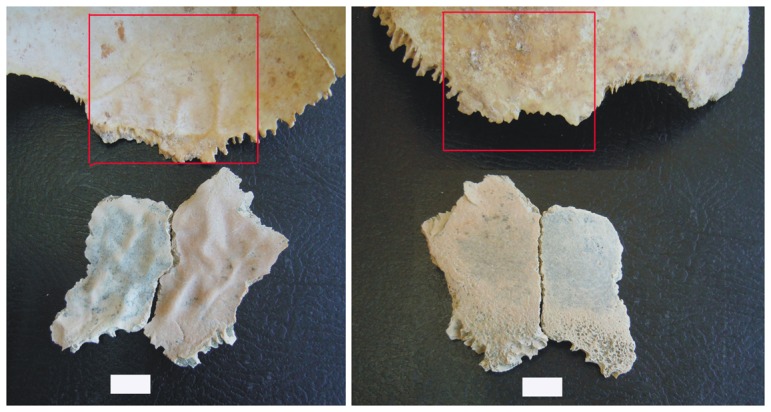
Endocranial (bottom left) and and ectocranial (bottom right) views of the OH 81 fossil (lower) compared to a parietal fragment of a modern human child in the same views. The parietomastoid suture and the lambdoid sutures form the inferior border of OH 81. Superior is up in both images, anterior is to the left in the lefthand image and to the right in the righthand image. Scale = 1 cm.

The exact relationship of porotic hyperostosis to various anemias is debated [1], [6]–[10] but current research hypothesizes its production in infants and young children through the combined effects of hypoferremia (induced by the ingestion of breast milk depleted in vitamin B_12_ or loss of access to vitamin B_12_ through weaning) and gastrointestinal infections [2]. Such an etiology fits with observations of OH 81, which consists of two refitting pieces of the posterior portion of a right parietal (one piece is 53.93 mm long [major axis] and 32.33 mm wide, the other 47.31 mm long [major axis] and 24.56 mm wide; refitted, OH 81 is 65.35 mm long anteroposteriorly and 53.38 mm wide superoinferiorly). All fracture edges of both pieces that comprise OH 81 were created prior to fossilization; adhering sediment had to be cleaned from the fracture edges and a small portion of the separated sutures of both fragments. Refit, OH 81 preserves a small segment of the lambdoid suture, the complete contour of the parietomastoid suture and the small anterior projection of bone that spans the parietomastoid and squamous sutures. Ectocranially, the specimen is truncated inferior to the unpreserved parietal tuber and temporal lines. Endocranially, the specimen preserves several digital impressions, and the overall cortical topography is similar to that of a very young modern human (*Homo sapiens*) child (Fig. 2). In addition, the presence of an incipient diploë on OH 81, which begins developing in modern humans around two years of age [11], prompts us to assign the specimen as a ∼2-year-old child. Further, the relative size and unfused state of the lambdoid and parietomastoid sutures correspond to the condition of modern humans of about two years of age [11]. Finally, taking into account that OH 81 derives from a smaller-brained hominin species than is *H. sapiens*, the relative morphology and size of the fossil parietal matches those of some estimated two-year-olds in a sample of XVIII^th^ century modern human crania from the necropolis of the Nuestra Señora de la Asunción Church (Chinchón, Madrid) (see **Methods**).

The presence of a porotic lesion on the ectocranial surface of OH 81 suggests the child from which the specimen derived possessed a digestive physiology adapted to the regular consumption of meat. This paleopathologically based conclusion supports previous hypotheses, based on stable carbon isotope and zooarchaeological and taphonomic data, of habitual carnivory by some Pleistocene hominins [12].

## Results

**Figure 3:**
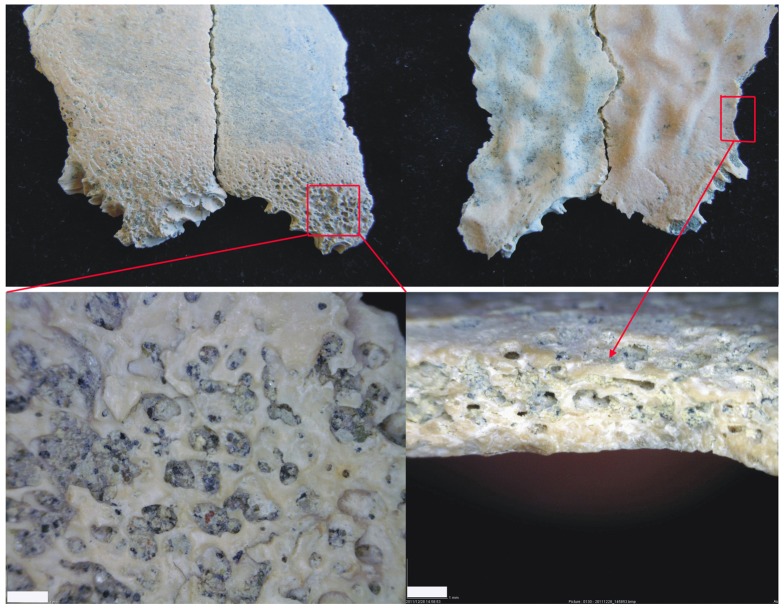
Ectocranial (top right) and endocranial (top left) close-up views of the OH 81 fossil, accompanied by magnifications of the porotic hyperostosis paleopathology as observed ectocranially (lower left) and edge-on at the diploic-table junction (lower right). Scale = 1 mm.

Both cortical surfaces of OH 81 are well-preserved, with neither showing any evidence of trampling, microabrasion or other mechanical alteration imparted either biostratinomically or diagenetically. However, a portion of the specimen's ectocranial rim, following the traces of parietomastoid and the lambdoid sutures is discolored and porous, with diploë exposed through the outer table in this area, with greatest expression superior to the parietomastoid suture (Fig. 3). Because only the outer table of OH 81 is thusly affected, we rule out diagenetic chemical alteration as the cause of these bone surface modifications. Further, eroding chemical alteration of cortical bone is completely absent on the rest of the specimens and in the larger fossil assemblage from which OH 81 derives (see **Methods**). Finally, the open sutures of OH 81 are sharp, unaffected by weathering and unmodified diagenetically, as would be expected if chemical alteration was the cause of the modified ectocranial surface observed on the specimen. Thus, we conclude that the origin of the porosity on the ectocranial surface of OH 81 is paleopathological.

The structure of the porous area of OH 81's ectocranial surface is in the form of round-shaped trabeculae that connect with the inner cancellous diploë of the specimen (Fig. 2 and 3), and is indistinguishable from occurrences in modern human crania diagnosed as specific forms of porotic hyperostosis [13], [14]. The diploë of OH 81 is also abnormally altered, with dilated medullary spaces due to the anomalous modification of internal collagen structure and lamellae.

A general diagnosis of porotic hyperostosis is usually applied in cases of porous enlargement of bone tissue, which can be caused by several types of disease or lesions. Some diseases modify the cranial periosteum but do not affect the tissues underlying the outer table. A common type of cranial porotic pathology, induced by the enlargement of hematopoietic marrow as a result of anemia, produces porous remodeling of the outer table. This type of porotic hyperostosis usually proceeds from the thinning of the external lamina and exposure of diploë, followed by hypertrophic radial growth of trabeculae. The enlargement of cancellous bone eventually creates an atrophic thickening of bone in the form of hair-on-end or comb-like structures [15]. This pathological pattern often affects a large area of the parietal bones (especially the parietal bosses), the occipital squama and, occasionally, the frontal bone [16]. Lesions that were active at the time an individual died show a porotic outer table or complete exposure of diploë. Healed lesions show infilling of the pores with new, dense bone, and remodeling of the lesions [17].

Although the macroscopic morphology of these porotic alterations is similar to those produced by other, related pathologies, Schultz [18] stresses important microscopic differences between the various distinct pathologies that present a macroscopic similarity to porotic hyperostosis pathologies. Specific pathologies that can be confused macroscopically with anemia-induced porotic hyperostosis include hemorrages (subperiostial hematomas) produced by scurvy, or inflammatory (periostitis, osteomyelitis) diseases [19].

Scurvy-related pathologies are relatively easy to diagnose, as the disease is associated closely with mastication and thus occurs most regularly in the cranium on maxillary and sphenoid bones [14], [20]. Atrophy of osteological tissue induced by scurvy only affects bone cortex and not internal bone structure [18]. This is because the widespread subperiosteal bleeding associated with scurvy prompts trabecular bone formation [19]. The trabeculae newly built in response to scurvy form short-bridged structures [18], a pattern of osteological porosity that is quite different from that of porotic hyperostosis produced by anemia, with its larger bridged structures [20]. Thus, because OH 81 clearly shows the exposure of the internal bone structure, with the disappearance of the outer table and *not just the deposition of trabecular osteological tissue on the cortical surface*, scurvy is ruled-out as the cause of the porotic hyperostosis of the specimen. In addition, the trabeculae of the exposed diploë of OH 81 are larger-bridged than the short-bridged pattern characteristic of scurvy-induced pathologies [18], [19].

Like scurvy, rickets is also capable of inducing porotic hyperostosis, but the porosity resulting from rickets is much less extreme than that created by anemia, eliminating any confusion in a differential diagnosis of the two diseases based on the intensity of osteological porosity [19]. Additionally, pathologies induced by inflammatory conditions, like periostitis, osteitis and osteomyelitis, display different microscopic porosity structure than is observed with porotic hyperostosis [15]. Because inflammatory diseases usually result in a combination of bone destruction as well as reactive bone formation in regions of necroticized tissue [14], the resulting bone pathologies present as rugged surfaces with spaced pitting and plaques of new bone; this is in contrast to porotic hyperostosis, where new bone only occurs in healed pathologies. Periostitis, specifically, can affect both the inner and outer structures of bone, with hypertrophic bone adopting a distinctive shape in which trabeculae are structured parallel to one another and oriented radially to the bone surface (although fibrous bone patterns are also documented). The newly built bone pattern is irregular and shows various structures that are the result of rapid growth associated with a high rate of remodeling [18], [19]. This pattern might thus appear similar macroscopically to that of the more advanced stages of of anemia-induced porotic hyperostosis, but is still dissimilar macroscopically to the earliest phase of porotic hyperostosis, which we argue is the condition for OH 81.

In this early phase, porotic hyperostosis induced by anemia shows a reduction of the structures of the red bone marrow modules, with trabeculae occurring tangentially to the outer table. Once the outer table is eroded, thin, flat squamous plates (osteophytes), in the form of atrophic trabeculae, are observable in their initial horizontal alignment [18]. This structure is readily apparent in OH 81 (Fig. 2 and 3), and because it is diagnostic of early porotic hypertostosis—occuring prior to extreme hypertrophy, when new cancellous bone presents as radially patterned trabeculae—it is imposible to mistake for pathologies induced by inflammatory conditions [19].

**Figure 4:**
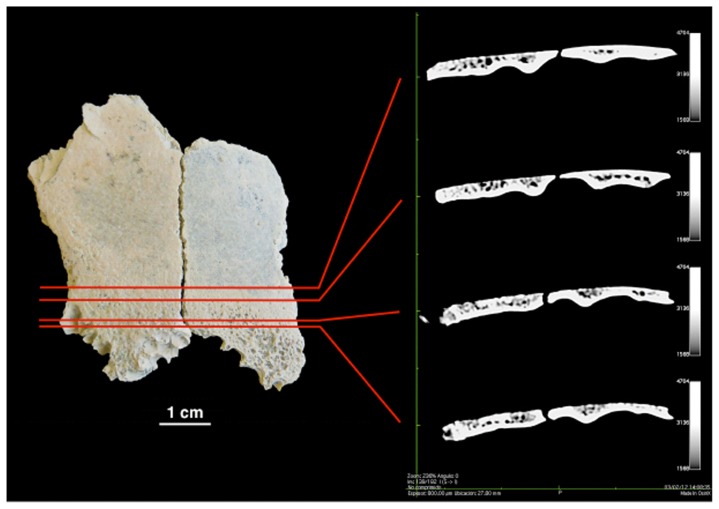
Ectocranial view of the OH 81 fossil (left) and a series of sagittal profiles of scanned sections of the specimen showing deterioration of outer table (top of each section) and exposure of diploë.

Several other diagnostic characteristics of anemia-induced porotic hyperostosis are apparent in the scanned images of OH 81. Fig. 4 illustrates the contrast in the progressively eroded outer table and trabecular diploic exposure between the least and most affected regions of the parietal. The upper two scan sections, on the right of the image, illustrate the basic radial pattern of trabeculae, typical of the early phase of anemia-induced porotic hyperostosis. The trabeculae at this early stage of pathological alteration do not yet form hypertrophic bone beyond the area previously covered by the outer table, but in more advanced stages (Schultz's [15] stages 2–3 and 3) they generate hypertrophy expressed as general swelling of cancellous bone. In addition, the typical polished marrow modules, asssociated with anemia-related porotic hyperostosis but not typically with pathology induced by inflammatory diseases are apparent on OH 81 (Fig. 3).

In sum, the combination of a modified outer table and hypertrophic diploë, as presented in OH 81, corresponds to stage 1 of Schultz's [15] paleohistopathological sequence of porotic hyperostosis. In stage 1, the porotic change to the parietal occurs most often in proximity to the lambdoid suture, and the external lamina of the ectocranial surface is reduced, whereas the lamina of the endocranial surface is unaffected [7], [15]. This description matches precisely the presentation for OH 81, which lacks thickening of the parietal but shows slight enlargement of the red bone marrow modules near the sutures (see Figure 16a of Schultz [15] for an example of porosity on the same locus as in OH 81). This same pattern of pathological modification has been observed on parietals of some modern humans from the Neolithic, Bronze Age and Middle Age archaeological records, where it has been routinely attributed to the effects of anemia [15], [21].

## Discussion

Walker et al. [2] argue that iron-deficiency anemia alone cannot explain the massive red blood cell production that causes the expansion of the marrow cavities in porotic hyperostosis, and that the overproduction of red blood cells seen in hemolytic and megaloblastic anemias is the most likely proximate cause of the pathology. Others suggest that porotic hyperostosis is related to hypoferremia in the presence of high pathogen loads and is thus not detrimental but instead advantageous in adapting to a heavily pathogenic environment [9]. However, porotic hyperostosis has also been documented among populations with a iron-rich diet [2], [21]. The relationship between porotic hyperostosis and hemolytic anemias, like sicklemia and thalassemia, has also been stressed, suggesting its linkage to malaria [22]. Given that porotic hyperostosis is often documented in human infants of roughly the same estimated age as OH 81 from regions free of malaria [7], we thus conclude that serious nutritional stress at a key phase in the development of the OH 81 individual was the most likely cause of the porotic hyperostosis observed on the fossil. Specifically, the individual likely suffered from a form of anemia related to a dietary deficiency of vitamin B_12_ (cobalamin) and B_9_ (folic acid) [2], [3]. Because of its specificity, this diagnosis stands as an important exception in the early hominin fossil record, where abundantly documented incidences of hypoplastic defects in tooth enamel indicate definitive physical stress in our ancestors but lack more specific etiology [23]–[26]. (Rarely, examples of dental caries are also known in some early hominin teeth [27]; there is a diagnosis of general periodontitis in *Australopithecus africanus* [28]; one of possible septicemia in the Nariokotome Boy *H. ergaster/erectus* skeleton [29]; possible brucellosis in *A. africanus* [30] and evidence of yaws or hypervitaminosis A in the KNM ER 1808 partial *H. ergaster/erectus* skeleton [31], [32].)

Although we cannot discard other causes for this pathology, we exclude prolonged nursing as a reason for it given that the specimen does not seem to indicate a development of the diploë and a size of the parietomastoid suture significantly larger than those observed on the crania of modern two-year old infants. Further, taking into account that early Pleistocene hominins experienced a more accelerated life history than do modern humans (see below), it is quite possible that the OH 81 specimens derive from an individual who was even younger than two-years-old when he died. Although iron deficiency anemia frequently develops in modern humans from communities that experience heavy intestinal parasite loads (and hominins were very likely exposed to a variety of intestinal parasites) the lack of proper documentation of porotic hyperostosis among apes [14], [19], [33], despite being exposed to a similar or even higher load of parasites [34], increases the likelihood that the anemic condition documented in OH 81 is dietary-related. Oesophagostrum (stomach worms) and intestine worms (nematodes and tape worms) in apes contain a varied zoonose [34]. Criba orbitalia has been documented in several primate taxa, but no cases of porotic hyperostosis are reported in the same study [33]. This emphasizes that even though criba orbitalia and porotic hyperostosis are often linked causally, there is actually no clear evidence upon which this interpretation of the inter-relationship between the two pathologies is based [2], [16], [21]. Further, porotic hyperostosis seems to develop from a prolonged (sometimes, chronic) condition as opposed to a temporary one such as iron-withholding by parasite infection [17]. Among modern populations infested by hookworms, there is no documented correlation between anemia and heavy loads of this parasite [17]. Additionally, hookworm infestation increases with host age, yet prevalence of anemia decrease through chilhood [17]. Last, the relative importance of pathogens in triggering porotic hyperostosis is evidenced by some foraging populations (such as the Hadza in northern Tanzania), in which parasitic infection, as a prolonged condition, has been documented in >10% of the population (27% of parasitemia in children) but yet no cases of porotic hyperostosis are known [35].

Thus, in sum, although parasites are sometimes definitely a contributing factor to prolonging anemic conditions, they are very unlikely the *cause* the cause of porotic hyperostosis. The only clinical studies in which a direct cause-and-effect relationship has been established for porotic hyperostosis, beyond the apparent correlations documented in some cases with pathogens [9] or iron-defficient diets [17], conclude that this pathology is caused by marrow hypertrophy in response to nutritional megaloblastic anemia [2]. Lack of B_12_ and B_9_ are the most common causes of megaloblastic anemia [2]. B_12_ defficiency compromises the immune system [2]. Parasites may decrease B_12_, but in people with normal B_12_ intake, parasite-associated loss of B_12_ is never enough to cause this pathology [2].

Due to its fragmentary nature, we cannot assign OH 81 to a particular genus or species of early hominin (see **Methods**), but, based on previous discoveries from the fossil's general temporogeographic context, reasonable choices include *H. habilis*, *H. ergaster/erectus* or *Paranthropus boisei*. Recent stable carbon isotope analyses of enamel, indicate that *P. boisei*, a highly craniodentally derived species known from Olduvai Bed II, was a specialized grass-eater [36]. But even specialized vegetarian primates, like extant *Gorilla*, require non-herbaceous dietary sources (e.g., coprophagia, insectivory) to culture the gut bacteria that can, in turn, synthesize cobalamin. Given the stone cutting tools and butchered animal bones that are associated directly [37] and penecontemporaneously with OH 81 (throughout East and South Africa) [38], meat was a more probable, source of vitamin B_12_ for early hominins—especially for reconstructed dietary generalists, like early *Homo*, also known from Bed II, Olduvai. If the OH 81 hominin was a member of the genus *Homo*, we conjecture further that he/she likely was a representative of *H. ergaster/erectus*, since the most recent occurrence of *H. habilis* at Olduvai Gorge is from the site of MNK (Mary Nicol Korongo), which is situated below SHK stratigraphically [37]; the only *Homo* fossils known to derive from above MNK stratigraphically, in Olduvai Beds II–IV, have been assigned to *H. ergaster/erectus*.

Together, these observations suggest that if OH 81 was still nursing at the onset of anemia, then his/her mother's diet was deficient in animal product. Alternatively, if OH 81 was being weaned at the onset of anemia, then it was the meat component of his own diet that was inadequate. The nutritional stress caused by weaning can lead to megaloblastic anemia, which frequently, in conjunction with gastrointestinal infections, produces diploic marrow hypertrophy, resulting in the ectocranial exposure of diploic trabeculae, typical of porotic hyperostosis [2]. For traditional people, weaning is critical phase in the life cycle, a time when the child not only loses passive immunity from his/her mother but when he/she is also exposed to new sources of potential infection and usually experiences decline in caloric intake, as well as significant nutrient deficiencies [39], [40]. High to very high frequencies of infant mortality are often documented for archaeological and extant populations of foragers and agropastoralists [41], [42]. Because of their greater rate of growth, infants require more iron and vitamins than other age groups [42], [43]. Brain maintenance and growth (drawing 80% of basal metabolic rate in infants) also requires high ntakes of protein, calories and water-soluble B vitamins (especially B_12_) [44]. Maternal breast milk is the ideal food during the first few months of life, but infant iron stores decline after six months of age [17], [43]. After this age, children enter a crucial period during which iron- and vitamin-rich solid foods should be introduced into their diets in order to maintain normal growth [41]. In addition, human milk is low in protein and after two years it can only satisfy about 50% of the infant's protein needs [44]. Because many modern children are often fed carbohydrate-based staples that contain little iron, cobalamin and folic acid, early childhood (especially weaning) is a critical period among foragers and non-industrialized food producers [41].

Malnutritionally derived porotic hyperostosis documented on the OH 81 early hominin fossil is significant for further elucidating the life histories of our African ancestors. If, as we estimate, OH 81 died from malnutrition when he/she was about two-years-old, then it might follow that his/her species engaged in a similar weaning schedule as do modern humans, which, on average, wean their offspring at 2.5 years of age [42], [44], beyond 2.5 years-old the healthy growth of the large modern human brain cannot be sustained on a diet based on milk [44]. Modern apes wean their offspring substantially later than do modern humans, usually between 4 and 7 years of age [43], [44]. Potentially, the hypothetical contrast between OH 81 and modern apes is even starker, given that all reasonable estimates conclude that growth in Early Pleistocene hominins advanced more rapidly than it does in modern humans [45], [46]. If these estimates of accelerated early hominin growth are correct, then the OH 81 hominin was probably even younger than two years old when he/she died. This accelerated growth would grant more support a nutritional deficiency in the mother's diet rather than weaning as the reason for the porotic hyperostosis documented on the OH 81 fossil. Alternatively, it could also indicate or a very precocious weaning compared to modern humans.

## Conclusions

The observation of porotic hyperostosis on the 1.5 Ma OH 81 child indicates early childhood was also nutritionally stressful for hominins, even well before the establishment of the sedentary, food-producing societies of the Holocene, and it also dispels the notion of invariable dietary affluence in the early Pleistocene. And, based on other pathological hominin fossils from more recent in time, the Pleistocene continued to be a stressful period for some hominin populations, in some places. For instance, cribra orbitalia, a specific form of porotic hyperostosis, affects one third of the Atapuerca (Spain) Middle Pleistocene Sima de los Huesos (Bone Pit) population [47].

Stratigraphically, OH 81 was recovered from the lower portion of upper Bed II of the Olduvai Formation, in which a geologically documented drying trend ultimately resulted in the disappearance of the Olduvai paleolake by late upper Bed II times [48]. This evolving climatic upheaval might have contributed to resource scarcity and nutritional stresses for Olduvai hominins. Thus, although archaeological evidence (in the form of stone tool butchery damage on ungulate fossils) [38] confirms the importance of meat-eating in human evolution, the same evidence weighed against nutritionally relevant paleopathological data, such as those for OH 81, reveals a more realistically nuanced picture of our prehistory.

Further broader relevance of anemia-induced porotic hyperostosis on OH 81 is its support for hypotheses that meat-eating was a fundamental, rather than marginal, aspect of some hominin diets during the early Pleistocene [38], [49]. There is disagreement, but a compelling body of data suggests [50]–[54] that extant chimpanzees (*Pan troglodytes*) might eat meat to compensate for seasonal shortfalls in protein and/or important micronutrients [55]. Despite the possibility that chimpanzees are at least seasonally obligate faunivores, porotic hyperostosis is virtually absent in these apes, our closest living relatives. In contrast, the pathology's relative prevalence in prehistoric hominins seems to indicate that a significant deriviation in hominin metabolic physiology from the ancestral condition occurred sometime after the late Miocene split between the hominin and panin lineages. The presence of anemia-induced porotic hypertostosis on the 1.5 Ma OH 81 hominin parietal, indicates indirectly that by at least the early Pleistocene meat had become so essential to proper hominin functioning that its paucity or lack led to deleterious pathological conditions. Actualistic studies on the ecology of scavenging show that this strategy is feasible only on a seasonal basis in some modern East African savannas and that it provides low flesh yields [56], [57]. A physiology adapted to the consumption of meat on a regular basis, as inferred for the species to which OH81 belonged, is in contradiction with this scenario, since it would not survive acquiring flesh so sporadically. This grants more support to the hypothesis that some hominins were actively engaged in hunting by 1.5 Ma.

Because fossils of very young hominin children are so rare in the early Pleistocene fossil record of East Africa, the occurrence of porotic hyperostosis on one, OH 81, suggests we have only scratched the surface in our understanding of nutrition and health in ancestral populations of the deep past.

## Methods

The SHK site, dated 1.5 Ma, was discovered in 1935, and is situated in the lower portion of upper Bed II of Olduvai Gorge, on the right bank of the Side Gorge, about 1 km east of the larger, upper Bed II site of BK [37]. The excavations carried out in 1953, 1955 and 1957 at SHK established two distinct areas in the site, the Main Site and a subsidiary site known as the Annex. The Main Site samples a fluvial channel and its overbank; deposits of an alluvial plain spreading away from the channel and its overbank are exposed at the Annex. The stratigraphic sequence (from bottom to top), as defined by Leakey [37] is: a >9 m thick chestnut brown clay; a conglomerate inside a channel, 1.8 m wide by 0.75 m deep; and a 2.4 m thick clayey tuff overlying the conglomerate in the channel and the clay laterally.

Leakey [37] hypothesized that the “hominin occupation level” on the surface of the clay of the Annex was penecontemporaneous with that of the Main Site, given that the brown clay upon which the level with archaeological materials occurs is the same in both locations and the same that the channel had eroded. Leakey [37] described 915 stone artifacts excavated from SHK, but faunal remains were not quantified or described, other than the mention of a complete *Hippopotamus gorgops* skull and the remains of a herd of *Antidorcas recki*. The number of artifacts reported was an underestimate, given that a large proportion of the “débitage,” as well as of the utilized materials, was discarded at the site. Analysis of only a selection of materials was originally carried out. Egeland and Domínguez-Rodrigo [58] reported 282 faunal specimens, representing 140 skeletal elements. The *A. recki* herd remains were not included in Egeland and Domínguez-Rodrigo's study because they are not curated at the National Museums of Kenya in Nairobi, which houses the materials from the Main Site. This herd remains also derive from a different stratigraphic provenience than the other faunal remains collected by Leakey [37]; the *A. recki* materials are from the overlying clayey tuff [59], while the rest of the SHK fauna excavated by Leakey is from the brown clay occupation level.

Our current excavations at SHK include expansion of Leakey's excavations at the Main Site, including in a portion of the channel containing a conglomerate and the overbank. Taphonomic analyses of the recently excavated faunal collection from the clay/conglomerate levels show that the formational history of the site is more complex than previously inferred [58], including the modification of bones by hominin butchers (work in progress). During our excavations in SHK, two subadult, refitting hominin parietal fragments (OH 81) were also discovered in the overlying clayey tuff, the same stratum from which Leakey excavated the remains of the *A. recki* herd.

Bone surfaces of OH 81 were first analyzed using a 60 W light and 15× hand lenses. Surfaces were then analyzed using a Motic™ binocular microscope, at magnifications of 20×–40× and an incorporated MC V3 digital camera, which transfers high-resolution images to a computer. Surface features of OH 81 were also observed under a handheld Dinolite AM413FVT™ digital microscope with magnifications of 10×–200× and analyzed using the microscope's (Dino Capture 2.0) software. Finally, OH 81 was scanned using a Philips Billance™ scanner with Phillips Brillance software 2.3.0. Each scan section is 800 µm. A total of 182 sections were produced producing images in DICOM format. Images were processed with OsiriX 3.8.1.

In order to estimate the ontogenetic age of the OH 81 composite fossil, we compared its size and morphology (variables included: state of diploic development, size of parietomastoid suture, fusion state of its sutures) to 14 XVIII^th^ century infant human crania from the necropolis of the Nuestra Señora de la Asunción Church (Chinchón, Madrid). Age estimates of the modern crania were based on tooth development and wear [60], [61]. Then we took the chord and arc dimensions of the parietals of aged crania and compared these to fragmented infant crania without associated dentition from the same modern sample and also to OH 81. By comparing OH 81 to some morphologically and developmentally similar crania from this modern sample, we could get an approximate estimate of the age of the individual to which the fossil belonged. However, there is still reason to conjecture that OH 81 was even younger than two-years-old when he/she died because most studies have concluded that some Early Pleistocene hominins developed more rapidly than do modern humans [46].

There are several subadult hominin cranial fossils, including OH 5 (*P. boisei*; 1.85 Ma) and OH 7 (*H. habilis*; 1.85 Ma), somewhat older than OH 81, but each died at a much more advanced age than did OH 81 (estimated to be ∼2-years-old at time-of-death, based on the state of its diploic development, the size of the parietomastoid suture, and the unfused state of its sutures, as compared with modern subadult human specimens of known age), which confounds their useful direct comparison to OH 81. The endocranial surface of OH 81, which lacks meningeal grooves and a developed sigmoid sulcus, is uninformative taxonomically. Ectocranially, the most taxonomically informative feature of the *P. aethiopicus*/*boisei* parietal is the squamous suture, which, even in juvenile specimens, typically evinces very strong overlap of the temporal and parietal bones, and presents marked ridges and finer *striae parietalis* that radiate superomedially from the parietal's temporal margin [62]–[64]. The very small portion of the posterior squamous suture preserved on OH 81 does not show this typical *Paranthropus* morphology. Even so, we conservatively allocate OH 81 to Hominidae gen. et sp. indet.
